# Serum amyloid A1 in combination with integrin αVβ3 increases glioblastoma cells mobility and progression

**DOI:** 10.1002/1878-0261.12196

**Published:** 2018-04-17

**Authors:** Ching‐Yu Lin, Shun‐Tai Yang, Shing‐Chuan Shen, Yi‐Chen Hsieh, Fei‐Ting Hsu, Cheng‐Yu Chen, Yung‐Hsiao Chiang, Jian‐Ying Chuang, Kai‐Yun Chen, Tsung‐I Hsu, Wan‐Chong Leong, Yu‐Kai Su, Wei‐Lun Lo, Yi‐Shian Yeh, Yudha Nur Patria, Hsiu‐Ming Shih, Che‐Chang Chang, Szu‐Yi Chou

**Affiliations:** ^1^ School of Medical Laboratory Science and Biotechnology College of Medical Science and Technology Taipei Medical University Taiwan; ^2^ Division of Neurosurgery, Shuang Ho Hospital Taipei Medical University Taiwan; ^3^ Department of Surgery, School of Medicine, College of Medicine Taipei Medical University Taiwan; ^4^ Graduate Institute of Clinical Medicine, College of Medicine Taipei Medical University Taiwan; ^5^ Comprehensive Cancer Center of Taipei Medical University Taiwan; ^6^ Graduate Institute of Medical Sciences, College of Medicine Taipei Medical University Taiwan; ^7^ Graduate Institute of Neural Regenerative Medicine, College of Medical Science and Technology Taipei Medical University Taiwan; ^8^ The PhD Program for Neural Regenerative Medicine College of Medical Science and Technology Taipei Medical University Taiwan; ^9^ Department of Medical Imaging Taipei Medical University Hospital Taiwan; ^10^ Department of Radiology, School of Medicine, College of Medicine Taipei Medical University Taiwan; ^11^ Research Center of Translational Imaging (TIRC), College of Medicine Taipei Medical University Taiwan; ^12^ Division of Neurosurgery, Department of Surgery Taipei Medical University Hospital Taiwan; ^13^ Graduate Institute of Translational Medicine College of Medical Science and Technology Taipei Medical University Taiwan; ^14^ Institute of Biomedical Sciences Academia Sinica Taipei Taiwan; ^15^ Neuroscience Research Center Taipei Medical University Hospital Taiwan

**Keywords:** glioblastoma multiform, integrin αVβ3, invasion, metastasis, serum amyloid A1

## Abstract

Glioblastoma multiforme (GBM) is a highly malignant type of brain tumor found in humans. GBM cells reproduce quickly, and the median survival time for patients after therapy is approximately 1 year with a high relapse rate. Current therapies and diagnostic tools for GBM are limited; therefore, we searched for a more favorable therapeutic target or marker protein for both therapy and diagnosis. We used mass spectrometry (MS) analysis to identify GBM‐associated marker proteins from human plasma and GBM cell cultures. Additional plasma and 52 brain tissues obtained from patients with gliomas were used to validate the association rate of serum amyloid A1 (SAA1) in different grades of gliomas and its distribution in tumors. Microarray database analysis further validated the coefficient of SAA1 levels in gliomas. The cellular mechanisms of SAA1 in GBM proliferation and infiltration were investigated *in vitro*. We analyzed the correlation between SAA1 and patients' medication requirement to demonstrate the clinical effects of SAA1 in GBM. SAA1 was identified from MS analysis, and its level was revealed to be correlated with the disease grade, clinical severity, and survival rate of patients with gliomas. *In vitro* cultures, including GBM cells and normal astrocytes, revealed that SAA1 promotes cell migration and invasion through integrin αVβ3 to activate the Erk signaling pathway. Magnetic resonance imaging and tumor region‐specific microarray analysis identified a correlation between SAA1 and GBM cell infiltration in patients. In summary, our results demonstrate that SAA1 in combination with integrin αV and β3 can serve as an indicator of high glioblastoma risk. We also identified the cellular mechanisms of SAA1 contributing to GBM progression, which can serve as the basis for future GBM therapy.

**Abbreviations**
CT: computed tomographic
DIH1: isocitrate dehydrogenase 1
EGFR: epidermal growth factor receptor
FPRL‐1: formyl peptide receptor‐like 1
GBM: glioblastoma multiforme
MMP9: matrix metallopeptidase 9
MRI: magnetic resonance imaging
MS: mass spectrometry
PTEN: phosphatase and tensin homolog
SAA1: serum amyloid A1
TCGA: The Cancer Genome Atlas
TMZ: temozolomide

## 1 Introduction

Gliomas are the most common tumors in the central nervous system. According to World Health Organization (WHO) guidelines, gliomas are divided into four grades (I–IV) based on pathological phenotypes; grade I and II gliomas are low‐grade gliomas, whereas grades III and IV (glioblastoma multiforme, GBM) are highly malignant types. GBM cells reproduce quickly and increase intracerebral pressure, causing headaches, vomiting, and drowsiness in patients. The standard treatment for patients with GBM includes surgical resection, radiation, and chemotherapy with temozolomide (TMZ); additional Avastin (bevacizumab) (antiangiogenesis) or gefitinib can be used to interrupt epidermal growth factor receptor (EGFR) signaling. However, the survival rate of patients with GBM is poor at 12–14 months. Appropriate GBM biomarkers are urgently required for early diagnosis and validation of interventions. Magnetic resonance imaging (MRI) or computed tomographic (CT) scans are used to identify GBM in patients with symptoms, but imaging examinations are costly and require special techniques in tertiary hospitals. Therefore, identifying a circulating indicator from patients' blood is crucial to enable early diagnoses and provide a therapeutic target.

Several genes associated with GBM development have been identified as representative molecular biomarkers, such as isocitrate dehydrogenase 1 (IDH1) mutations, chromosome 1p19q co‐deletion, O6‐methylguanine‐DNA‐methyltransferase promoter methylation, phosphatase and tensin homolog (PTEN) mutations, and EGFR variant III (EGFRvIII) amplification (Hill *et al*., 2003; Rich *et al*., 2005; Wang *et al*., 2015). Genetic and molecular variability in patients with GBM, however, causes different outcomes and resistance levels after therapy and can lead to severe relapse. Recently, noncoding RNA, omics studies, and proteomic analyses of GBM extracellular vesicles have been helpful in identifying additional associated biomarkers and potential treatment strategies for GBM (Ivo D'Urso *et al*., 2015; Kros *et al*., 2015; Touat *et al*., 2015).

Serum amyloid A1 (SAA1) is an acute‐phase high‐density lipoprotein secreted by the liver in response to infection and tissue injury. Plasma levels of SAA1 are elevated following injury, inflammation, and brain trauma, as well as cancer (Lu *et al*., 2014; Milan *et al*., 2012; Sung *et al*., 2011; Villapol *et al*., 2015). A transcriptomic analysis of patients with cervical squamous cell carcinoma revealed that SAA1 levels were associated with tumor size, lymphatic metastasis, and cancer cell invasion (Peng *et al*., 2015; Ren *et al*., 2014). SAA1 has also been proposed as a lung cancer biomarker, and patients with lung cancer who have higher levels of plasma SAA1 have less favorable outcomes (Kim *et al*., 2015; Milan *et al*., 2012; Sung *et al*., 2011). Patients with higher SAA1 levels are resistant to treatment with gefitinib, an EGFR tyrosine kinase inhibitor (Garrisi *et al*., 2011; Milan *et al*., 2012). However, the role of SAA1 in gliomas remains unclear. Knebel *et al*. (2013) revealed SAA1 to have a dual role in glioma migration regulation, although the mechanism is unclear, and upregulated SAA1 levels in human glioblastomas were suggested to create a high‐inflammation microenvironment (Knebel *et al*., 2017).

In this study, we first used mass spectrometry (MS) to determine the elevated levels of SAA1 in the plasma and GBM cell secretions of two patients. Both the plasma and tumor levels of SAA1 were positively correlated with the grade and severity of gliomas. Both intracellular and secreted forms of SAA1 promoted astroglia migration and invasion, with integrin αVβ3 functioning as a crucial downstream effector. Results from both MRI and tumor region‐specific microarray assessments further validated the distribution and effect of SAA1 in GBM infiltration. In summary, our findings suggest that SAA1 levels in plasma are an indicator of GBM and a potential therapeutic target in GBM.

## 2 Materials and methods

### 2.1 Human tissues and plasma samples

Blood plasma samples from both the glioma and normal cohorts were obtained from the Taipei Medical University Hospital Biobank and Shuang Ho Hospital, Taiwan. Brain tissues were obtained from the Taipei Medical University Biobank. This study was approved by the Ethical Committee of Taipei Medical University (institutional review board (IRB) approval no.: B201501003, 201204002, 201402018, 201503039, 201603086; biobank no.: B201202001; protocol approval no.: 201006011) for patient‐derived GBM cells. Informed consent was obtained from all patients whose tissues were used. The demographic characteristics of patients are presented in Tables 1 and 2.

**Table 1:** Demographic characteristics of control normal subjects and glioma patients investigated in the current study. SD, standard deviation; WHO, World Health Organization

	Normal subjects	Mass discovery study	Validation study
		Glioma subjects	Glioma subjects
Total subjects	12	12	43
Male, *n* (%)	4 (33)	4 (33)	23 (53)
Age, years, mean (SD)	60.1 (18.1)	60.2 (18.7)	53.3 (17.9)
WHO grade, *n* (%)			
Grade I	–	–	16 (37.2)
Grade II	–	–	7 (16.3)
Grade III	–	–	7 (16.3)
Grade IV	–	12 (100)	13 (30.2)

**Table 2:** Basic characteristics of glioma patients with IHC scores of high (≥2) and low (≤1) serum amyloid A1 (SAA1) expression. SD, standard deviation; WHO, World Health Organization

	SAA1 score		*P*‐value
	≤1	≥2	
Age, years, mean (SD)	53.7 (16.3)	46.4 (24.7)	0.2379
Gender, *n* (%)			
Female	16 (53.3)	11 (50.0)	0.8121
Male	14 (46.7)	11 (50.0)	
WHO grade, *n* (%)			
I	18 (60.0)	2 (9.1)	<0.0001***
II	11 (36.7)	1 (4.6)	
III	0 (0)	7 (31.8)	
IV	0 (3)	12 (54.6)	

***P <* *0.001, comparison of grades I and II.

### 2.2 Procedure of biomarker identification

Plasma protein concentration was quantified through the BCA protein assay (Life Technologies); 400 μg of plasma protein was subjected to albumin immunodepletion, followed by a depletion efficiency assessment through sodium dodecyl sulfate–polyacrylamide gel electrophoresis (SDS/PAGE) and stained with Protein Staining Reagent (TOOLS, New Taipei City, Taiwan), then trypsin digestion using an In‐Solution Tryptic Digestion and Guanidination Kit (Thermo Scientific, Waltham, MA, USA). Trifluoroacetic acid was added to stop the trypsin digestion reaction. Samples were then desalted with ZipTip C18 tip (ZTC18S960, Merck Millipore, Darmstadt, Germany) and dried in SpeedVac SC110 concentrators (Savant Instrument, Hyderabad, India). The samples were analyzed by a NanoAquity UPLC system (Waters, Milford, MA, USA) coupled to an Orbitrap Elite mass spectrometer (Thermo Electron, Waltham, MA, USA) in positive ion mode. The acquired raw data were qualitatively and quantitatively processed using PEAKS Studio (PEAKS 7, Bioinformatic Solution, Waterloo, ON, Canada), and the protein intensity quantification was conducted using a label‐free quantification method. More details are provided in the Supporting Information.

### 2.3 Cell studies and stable cell line generation

U87MG (U87, ATCC: HTB‐14) and SVG‐p12 cells (SVG, ATCC: CRL‐8621) were obtained from the Bioresource Collection and Research Center, Taiwan. GBM patients' cells were surgically isolated by neurosurgeons (IRB Protocol approval no.: 201006011). Constructs encoding for nontargeting (scrambled, control) and two different SAA1‐specific short‐hairpin (sh) RNA (shRNA) plasmids (plasmid no. TRCN0000373397 and TRCN0000373398 obtained from the Academia Sinica RNAi Core Facility and designated as shSAA1‐1 and shSAA1‐2, respectively) were introduced into cells to generate SAA1‐knockdown cell lines, and a scrambled nucleotide plasmid was used as a control. Integrin αV and β3 plasmids were provided by Y. Takada (Department of Dermatology, UC Davis, USA). A wound‐healing migration assay was performed following previously published protocols (Liang *et al*., 2007; Rodriguez *et al*., 2005). The regions of interest were photographed and analyzed by ImageJ. For transwell migration, cells were seeded in a 24‐well culture insert with 8.0‐μm pores (Falcon‐353097); media were placed in the lower chamber to induce cell transmigration, and Matrigel^®^ was used for an invasion assay. Migrated cells were fixed with 4% paraformaldehyde and stained with 0.5% crystal violet solution. Cells passing through the chamber were photographed with a Leica DMIL LED Microscope and a charge‐coupled device camera. Nine different fields in each chamber were chosen for quantification, and three to five repetitions were conducted.

### 2.4 Protein immunoblotting

Plasma proteins and cell lysates were separated using SDS/PAGE. Immunoblotting was conducted through overnight incubation of membranes with an SAA1 antibody (Ab) (GTX20687, GeneTex, Irvine, CA, USA), integrin αV (#4711, Cell Signaling Technology, Danvers, MA, USA), integrin β3 (#13166, Cell Signaling Technology), FPRL‐1 (GTX130627, GeneTex), matrix metallopeptidase 9 (MMP9) (GTX23159, GeneTex), actin (AB5220, EMD Millipore), and α‐tubulin (NB100‐690, Novus Biologicals, Littleton CO, USA), followed by incubation with corresponding secondary Ab. Image intensity was quantified using imagej (Bethesda, MD, USA).

### 2.5 SAA1 immunohistochemical staining

Tissues were incubated with a SAA1‐specific Ab (16721‐1‐AP, Proteintech Group), followed by avidin–biotin complex conjugation and 3,3′‐diaminobenzidine development (PK6101, Vector Laboratories, Burlingame, CA, USA) (Chou *et al*., 2005). Hematoxylin and eosin staining was used for counterstaining. A semiquantitative score was applied to describe the distribution and intensity of SAA1 staining (0 = negative, 1 = weak, 2 = moderate, 3 = strong, and 4 = strong and widely distributed).

### 2.6 RNA extraction and microarray assay

Total RNA from cancer tissues was prepared using a mirVana miRNA Isolation Kit (Invitrogen, Waltham, MA, USA) according to the manufacturer's instruction. The quantity and quality of the extracted RNA were evaluated with an RNA 6000 Nano LabChip on an Agilent 2100 Bioanalyzer (Agilent Technologies, Palo Alto, CA, USA). Total RNA (500 ng) was amplified and labeled with an Agilent Quick Amp Labeling Kit according to the manufacturer's instructions. A cyanine 3‐CTP‐labeled cRNA sample (1.65 μg) was used for 17‐h hybridization at 65 °C by using a one‐color Agilent 60‐mer whole human genome array kit or whole rat genome array kit (Agilent Technologies) containing >40 000 unique genes and transcripts. The hybridized microarrays were washed and then scanned using an Agilent DNA microarray scanner. Signal intensities were quantified from the scanned image by using agilent's feature extraction software version 9.1 (Agilent Technologies). The quantified signal intensities were corrected for background intensities using the normexp method, followed by quantile normalization across arrays using the limma software package (Ritchie *et al*., 2007). To filter out noninformative probes, we removed positive and negative control probes and probes whose overall variance of intensity values were less than the median of the variances (Talloen *et al*., 2010). The reported microarray data were deposited in the Gene Expression Omnibus database (http://www.ncbi.nlm.nih.gov/geo).

### 2.7 Statistical analysis

Continuous variables are expressed as the mean and standard deviation and were analyzed using Student's *t*‐test. The Mann–Whitney *U*‐test was used for assessing protein relative abundance and intensity differences between calculated immunoblotting data of the control and GBM groups. The Kruskal–Wallis test was used to analyze blotting signal differences among the four stages of GBM. Categorical variables were analyzed using a chi‐squared test; however, when the frequency of any cell was less than five, Fisher's exact test was used. Kaplan–Meier curves and log‐rank tests were applied to estimate the differences in survival rates between groups with high SAA1 levels and those with low SAA1 levels. Data were downloaded from Betastasis (http://www.betastasis.com), including data regarding brain tumor subtypes and the expression levels of SAA1, integrin αV, integrin β3, and integrin β8; we performed a survival analysis through a univariate analysis method. Data processing and statistical analyses were performed using sas 9.4 (SAS Institute, Cary, NC, USA) and the graphpad prism 6 statistical software package. For cell studies, one‐way ANOVA was conducted to determine the difference between groups and a *t*‐test was conducted to compare two samples. A *P* value of <0.05 was considered statistically significant.

All other details concerning the materials and methods used in this study are provided in the Supporting information.

## 3 Results

### 3.1 MS analysis reveals increased SAA1 in GBM patients' plasma and glioma cell medium

**Figure 1:**
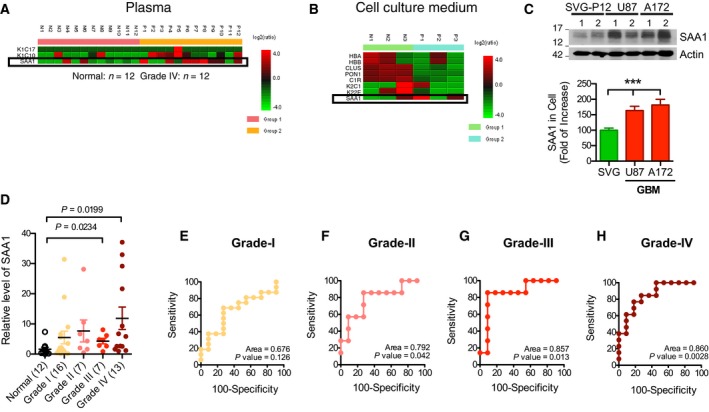
Plasma level of SAA1 is positively correlated with glioma malignancy. MS analyses of (A) plasma from patients with GBM and (B) culture medium of GBM cells. Levels of SAA1 were higher in both the plasma from patients with GBM and the culture medium of GBM cells. (C) Protein level of SAA1 in a normal human astrocyte, SVG, and two GBM cell lines, U87 and A172 (***P <* *0.001, by *t‐*test). The numbers 1 and 2 above the immunoblot scans represent two independent preparations of protein from cultures. (D) Plasma levels of SAA1 in cohorts and four glioma grades. (E–H) ROC curve of plasma SAA1 levels in glioma grades I–IV. Levels of SAA1 in plasma were specific and sensitive to glioma grades III (G) and IV (H).

Plasma samples from 12 patients with GBM and 12 normal individuals were analyzed through MS for biomarker discovery (Table 1). Three plausible proteins from GBM patients' plasma were identified: haptoglobin, SAA1, and serpin peptidase inhibitor‐clade A‐member 3 (Fig. 1A). Cultured media from the GBM cell line—U87—and normal human astrocytes—SVG—were also analyzed, and eight proteins were identified (Fig. 1B), including SAA1. Protein analysis confirmed the elevated expression of SAA1 in different GBM cell lines including U87 and A172 (Fig. 1C).

An additional 43 blood samples from four different grades of gliomas (WHO grades I–IV) were examined using protein blotting. The plasma levels of SAA1 in the control cohort and grades I and II were relatively low (Fig. 1D). However, SAA1 in the GBM samples was 7.2‐fold higher than that in the control cohort (Fig. 1D). The sensitivity and specificity of SAA1 to the glioma grades were positively associated with glioma grades III and IV, as indicated by a receiver operating characteristic (ROC) curve (Fig. 1G,H), but not with grade I or II (Fig. 1E,F).

### 3.2 SAA1 expression is inversely correlated with overall patient survival

**Figure 2:**
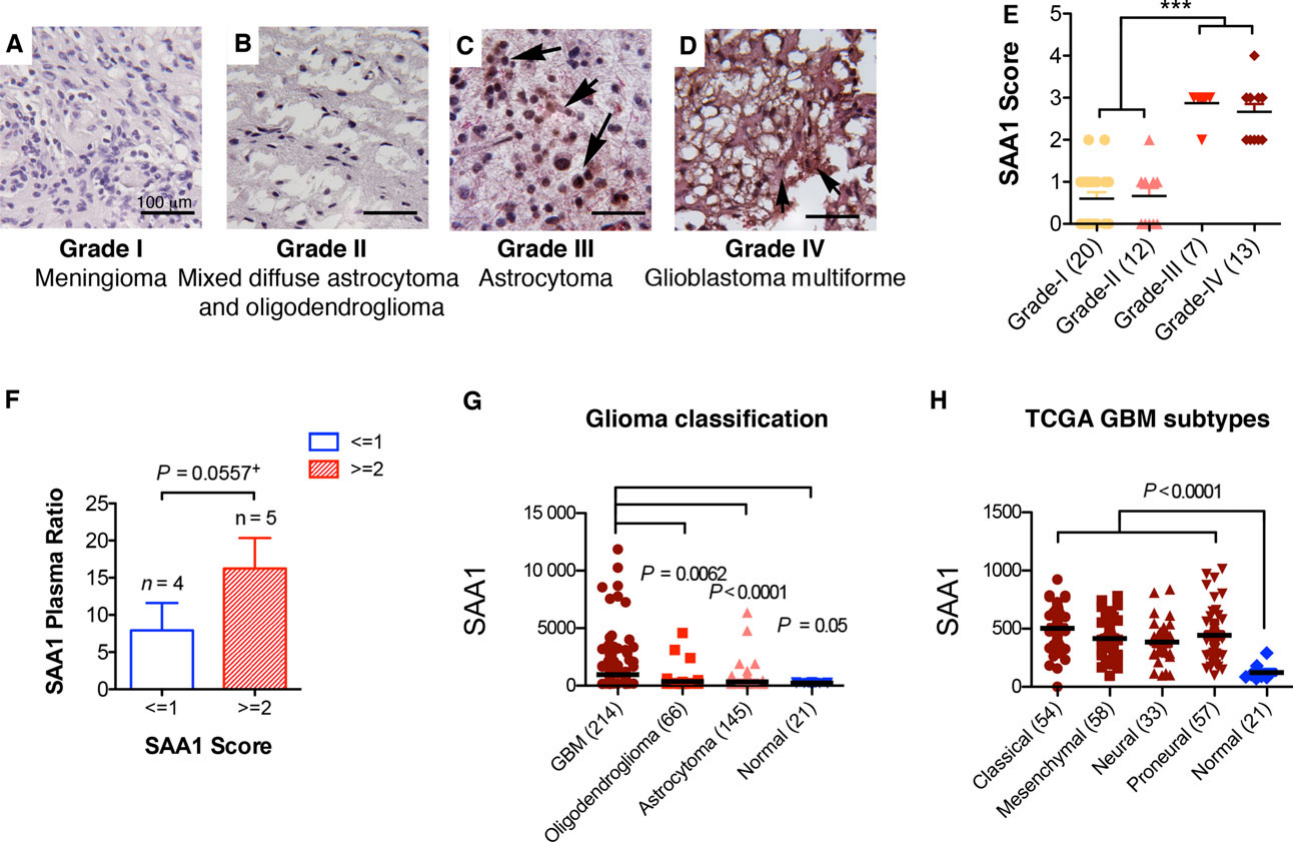
Serum amyloid A1 in human brain glioma tissues is associated with tumor malignancy and patient mortality. (A–D) Immunohistostaining of SAA1 in human glioma grade I–IV tissue sections. (E) SAA1 staining scores in different grades of glioma sections (***P <* *0.001 using the Kruskal–Wallis test). (F) Plasma levels of SAA1 in patients with low (≤1) or high (≥2) IHC staining scores. Microarray database (Betastasis) analysis of SAA1 expression in different gliomas based on (G) WHO histological classification and (H) GBM TCGA subtypes (*P* value between groups is given in the figure).

The expression and distribution of SAA1 were determined by immunohistochemical (IHC) staining using an SAA1‐specific Ab; 52 brain tumor tissues including four different glioma grades were analyzed (Table 2). The immunoreactivity of SAA1 was scored 0 for non‐SAA1 signals and scored 1–4 on the basis of the SAA1 staining intensity and distribution (score definition is provided in the Materials and methods section). The immunoreactivity of SAA1 in glioma grade I and II brain tumors was generally sparse and weak with a score of 0 (Fig. 2A,B). Only two of 20 cases in grade I and one of 12 in grade II demonstrated an SAA1 signal intensity of ≥2 (Fig. 2E). By contrast, the immunoreactivity of SAA1 in grade III and IV tumors was strongly manifested as a fiber structure in cytosol (Fig. 2C,D). In total, 7 of 7 cases (100%) in grade III and 13 of 13 cases (100%) in grade IV brain tumors demonstrated a signal intensity of ≥2 (Fig. 2E, also see Fig. S1). Moreover, 80% of patients with high (signal ≥ 2) SAA1 expression were grades III or IV, and more than 95% of patients with low (signal ≤ 1) SAA1 expression belonged to grades I and II (Table 2). The distribution of age and sex was not significantly different between patients with high and low SAA1 expression. In addition, among a total of nine patients whose plasma and tissue SAA1 levels could be compared, plasma SAA1 levels were positively correlated with tumor SAA1 labeling (Fig. 2F). The gene expression of SAA1 in patients with GBM (214 cases) was at least fourfold higher than that in patients with oligodendrogliomas (66 cases) (*P *=* *0.0062), patients with astrocytomas (145 cases) (*P *<* *0.0001), and normal patients (21 cases) (*P *=* *0.05) (Fig. 2G). Four TCGA (The Cancer Genome Atlas) GBM subtypes, namely classical, mesenchymal, neural, and proneural types, all expressed higher levels of SAA1 than those observed in normal patients (Fig. 2H) (*P *<* *0.0001) (data were obtained from Betastasis). Overall, our results suggest that the plasma level of SAA1 is positively correlated with tumor SAA1 expression, and peripheral SAA1 levels can thus be considered an indicator of glioma malignancy.

### 3.3 Tumor levels of SAA1 are associated with clinical diagnosis and treatment of glioma patients

To elucidate the association between SAA1 and the severity of patients' clinical status, patients' treatment histories were compared with their brain pathological analyses and SAA1 IHC scores. Patients who received neurological surgery also took dexamethasone (DEXA) or Rasitol as drug therapy to counteract the development of edema, and others underwent aggressive chemotherapy with TMZ after surgery. Among the studied patients, 71.8% who received DEXA, Rasitol, or both had low SAA1 IHC staining scores (score ≤ 1, Table 3). By contrast, 63.6% of patients who did not receive DEXA or Rasitol belonged to the group exhibiting high SAA1 expression (score ≥ 2, Table 3). Patients who received TMZ belonged to the group exhibiting high SAA1 expression (score ≥ 2, Table 3). Most of the patients with low SAA1 expression (score ≤ 1, Table 3) had not received TMZ.

**Table 3:** Medication and serum amyloid A1 (SAA1) IHC scores among different grades of glioma patients. DEXA, dexamethasone; TMZ, temozolomide

	SAA1 score		*P* value
	≤1	2+	
DEXA			
No	5 (41.7)	7 (58.3)	0.0889
Yes	27 (71.1)	11 (29.0)	
Rasitol			
No	23 (60.5)	15 (39.5)	0.4973
Yes	9 (75.0)	3 (25.0)	
DEXA + Rasitol			
None	4 (36.4)	7 (63.6)	0.0306*
Either/Both	28 (71.8)	11 (28.2)	
TMZ			
No	32 (74.4)	11 (25.6)	0.0003***
Yes	0 (0)	7 (100.0)	

*P <* *0.05; ***P <* *0.001, compared to the untreated group.

In summary, our findings indicate that SAA1 expression levels in tumor cells are positively correlated with glioma grade, disease severity, and patient mortality.

### 3.4 Glioma cells with high SAA1 expression demonstrate stronger motility and invasive capability

**Figure 3:**
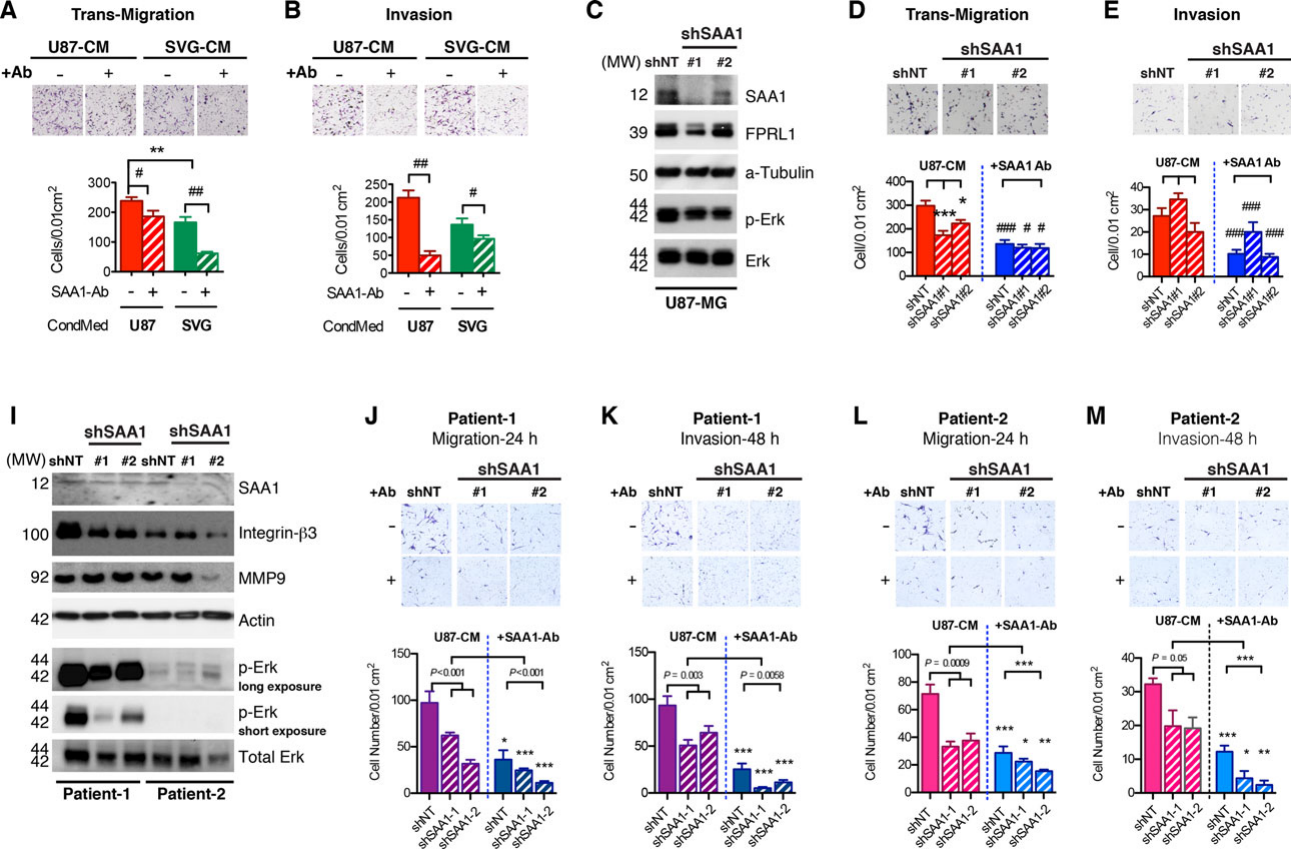
Serum amyloid A1 modulates glial cell migratory and invasive properties. Cell migratory and invasive properties were analyzed through a transwell array. Migrated cells were photographed and quantified. (A,B) U87 cells migrated (−Matrigel) and invaded (+Matrigel) upon U87‐CM or SVG‐CM treatment. SAA1‐specific Ab was pretreated (+Ab) with different CMs to chelate extracellular SAA1. Both the migration and invasion rate of U87 were reduced in SAA1 reduction medium (#*P *<* *0.05, ##*P *<* *0.01, compared with the Ab‐pretreated groups; **P <* *0.01 compared U87‐CM and SVG‐CM). (C) Two independent SAA1‐knockdown U87 cell lines—shSAA1‐1 and shSAA1‐2—were generated; shNT was used as control. The protein levels of SAA1, receptor (formyl peptide receptor‐1), and pErk in the two cell lines were analyzed. Alpha‐tubulin was taken as the loading control. (D–E) The number of migrating cells was reduced in shSAA1‐1 and shSAA1‐2, but the invaded cell members were not affected. Blocking extracellular SAA1 reduced U87 cell migration and invasion (*P <* *0.05, **P <* *0.01 compared with shNT; #*P *<* *0.05, ##*P *<* *0.01, compared with the Ab‐pretreated groups). Two patient‐derived GBM cell lines were applied; cells from patients 1 and 2 were transfected with scrambled nucleotide as the control or shSAA1‐1 and shSAA1‐2. (I) Protein levels of SAA1, integrin β3, MMP9, and pErk were reduced following SAA1 knockdown in patients. The migratory activity and invasive ability of different cells upon CM or SAA1‐Ab pretreatment (+Ab) were analyzed. Migration rates (J, L) and invasive ability (K, M) in both cell lines were reduced following SAA1 knockdown, which were suppressed further when the cells were treated with CM subjected to SAA1‐Ab pretreatment (*P <* *0.05; **P <* *0.01; ***P <* *0.001, compared with CM treatment, as determined through one‐way ANOVA).

Human recombinant SAA1 had been reported to promote GBM T98G cell migration (Knebel *et al*., 2013). The current study also noted a loose tissue structure in strongly labeled SAA1 areas (Fig. 2C,D). To investigate the role of SAA1 in GBM tumor progression, we tested the effects of both intracellular SAA1 and extracellular SAA1 on cell migration and invasion by using a transwell assay. A conditioned medium (CM) from U87 contains many proteins including SAA1; to validate the contribution of SAA1, a CM was depleted of SAA1 by using a SAA1‐specific Ab. The extracellular SAA1 from the U87 CM (U87‐CM) was placed in the lower chamber of the transwell assay system to induce cell migration and invasion. The SAA1‐specific Ab was pretreated to chelate with SAA1 in the U87‐CM. Fewer cells migrated through the transwell (Fig. 3A) or Matrigel^®^ (Fig. 3B) system in the SAA1‐depleted U87‐Matrigel‐CM compared to the U87‐SAA1‐Ab‐pretreated group and U87‐CM. Fewer migrated cells were found in the SVG‐CM group due to the lower amount of SAA1 in SVG‐CM (Fig. 3A,B).

Next, shRNA were applied to reduce the expression of SAA1 in U87 cells. Two independent SAA1‐knockdown cell lines were generated, namely shSAA1‐1 and shSAA1‐2, which reduced the expression of SAA1 (Fig. 3C), and a scrambled nucleotide shRNA (shNT) was transfected as the control. The cell proliferation rates were similar after the reduction in SAA1 (Fig. S2A). The migratory activity was significantly reduced in the two shSAA1 cell lines, with a stronger suppressive effect observed when the extracellular SAA1 was blocked (Fig. 3D). By contrast, the invasive ability was not affected after the silencing of the intracellular SAA1, but the invasive ability was suppressed when the extracellular SAA1 was chelated in culture (Fig. 3E). Our findings thus suggest that both intracellular SAA1 and extracellular SAA1 contribute to cell migration and that extracellular SAA1 is essential for extracellular matrix breakdown during invasion.

Recombinant nonglycosylated SAA1 had been reported to have a controversial role in promoting cell migration in different GBM cell lines (Knebel *et al*., 2013). To further elucidate the role of SAA1 in cell migration, we established two individual GBM primary cell lines obtained from clinical patients and transfected them with shSAA1‐1 and shSAA1‐2, respectively, to generate SAA1‐knockdown cell lines (Fig. 3I). The proliferation rate was not changed after SAA1 expression knockdown (Fig. S2B,C). The migratory activities of the cells obtained from patients 1 and 2 were reduced following intracellular SAA1 and extracellular SAA1 knockdown with shRNA and a specific Ab (Fig. 3J,L). Similar results were found in invasion studies; cells expressing lower SAA1 demonstrated lower invasive ability (Fig. 3K,M). Removing extracellular SAA1 suppressed the invasive activity of GBM cells obtained from both patients (Fig. 3K,M). We also investigated migratory capacity through a wound‐healing assay to obtain a temporal correlation. Cells were photographed after 0, 24, and 48 h and cultured with either a serum‐free (SF) medium or the U87‐CM. The open area was reduced with time in control cells cultured with the SF medium, and migration was accelerated when the cells from both patients were cultured with the U87‐CM (Fig. S2D,E). This migratory ability was successfully blocked when SAA1 was depleted in both patients' cells (Fig. S2D,E). These results thus confirm that migratory and invasive capacities are controlled by SAA1. SAA1, as a paracrine agent, induces tumor cell migration and invasion, which can consequently escalate the malignant grade of gliomas. We also tested downstream signaling activation. Among the different migration pathways, we determined that Erk signaling axis (phosphorylated Erk‐pErk) was reduced following SAA1 knockdown in U87 and the two patients' cell lines (Fig. 3C,I).

**Figure 4:**
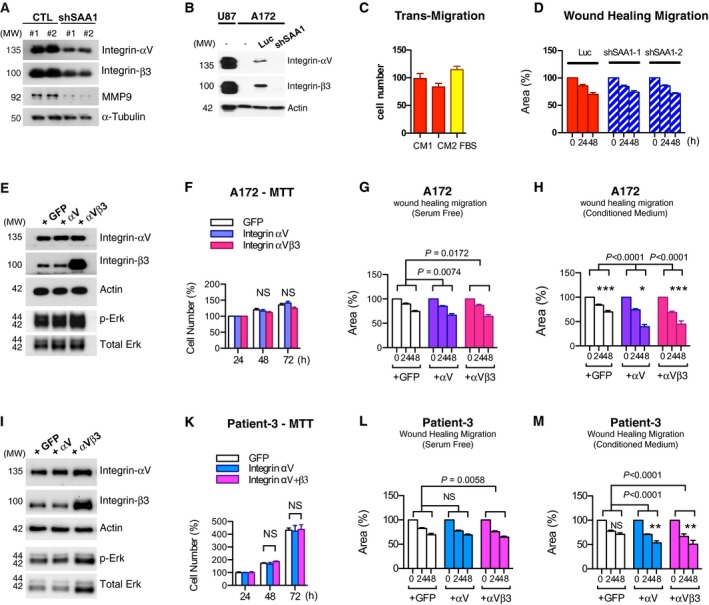
Integrin αV and β3 are critical signal molecules responsible for SAA1 regulating cell motility. (A) Migration‐related proteins—integrin αV, integrin β3, and MMP‐9—were reduced in SAA1‐knockdown U87 cell lines. (B) Levels of integrin αVβ3 in A172 cells were markedly reduced compared with those in U87 cells under different conditions including no transfection, transfection with scrambled nucleotides, or shSAA1. (C) Migratory ability of A172 cells was low in CM and FBS treatments in a transmigration assay. (D) Migration speed did not differ between control and shSAA1 cell lines in a wound‐healing migration assay. Integrin αV or αVβ3 plasmids were co‐transfected into (E) A172 and (I) a patient‐derived GBM cell line—Patient 3—to increase intracellular integrin αV or integrin αVβ3 together; a GFP was transfected as a control. Cell proliferation rates were similar in (F) A172 and (K) Patient 3 cell lines. Cell migratory activities were increased in A172 overexpressing both integrin αV and αVβ3, compared with GFP, when cultured in (G) SF medium and (H) CM containing extracellular SAA1. The migration activities upon CM were higher than that under the SF condition. Similar results were identified in Patient 3 cell line. Cell migratory activities were increased when overexpressing integrin αV and αVβ3 in SF medium (L) and CM (M) (*P <* *0.05, **P <* *0.01, ***P <* *0.001 between same cell line cultured in CM and SF. NS: No significant difference).

A study suggested that A172 glioma cells respond to SAA1 differently (Knebel *et al*., 2013). To clarify the contribution of SAA1 to the migration process, we performed additional mechanistic studies. We detected the protein levels of migration‐ and invasion‐related cellular molecules—integrin αV, β3, and MMP9—and found that these molecules were significantly reduced when SAA1 knockdown was induced in both patients' cells and U87 cells (Figs 3I, 4A). A172 did not respond to CM containing SAA1 or fetal bovine serum (FBS) in both the transmigration system (Fig. 4C) and wound‐healing migration test (Fig. 4D). Together, these findings suggest that some key components required for SAA1‐mediated migration are not present in A172 cells. A172 cells expressed high levels of SAA1 (Fig. 1C) but contained extremely low levels of integrin αV and β3 compared with U87 (Fig. 4B). Reintroducing integrin αV or integrin αV and β3 together into A172 cells increased the intracellular integrin αV and β3 levels (Fig. 4E) but not changed the cell proliferation rate (Fig. 4F); a green fluorescent protein (GFP) was transfected as the control (Fig. 4E,F). Next, we analyzed the migratory activity of A172 by performing a wound‐healing migration assay. We found that the migratory activity of A172 increased significantly when integrin αV was overexpressed alone or together with integrin β3 in the SF medium (Fig. 4G) and SAA1‐containing CM (Fig. 4H). The migratory activities of different cell lines were higher in the CM treatment compared with the SF medium. To confirm this finding, we also compared the integrin expression levels in U87, A172, and GBM cell lines obtained from different patients. We identified a GBM cell line obtained from one patient—Patient 3—to be similar to A172, which exhibited low integrin αV and β3 expression levels (Fig. S3). GFP, integrin αV, and β3 were transfected into Patient 3 GBM cells (Fig. 4I). The proliferation rate of cells was not changed by integrin or GFP overexpression (Fig. 4K) and, crucially, migratory activities were increased by overexpressed integrin αV and β3 (Fig. 4L). Extracellular SAA1 in the CM increased migratory activity, especially in cells with overexpressed integrin αV and β3 (Fig. 4M). In addition, the pErk signaling pathway was activated when integrins were overexpressed in A172 and Patient 3 cells (Fig. 4E,I).

### 3.5 Tumor levels of SAA1 and integrins are associated with the mortality of glioma patients

**Figure 5:**
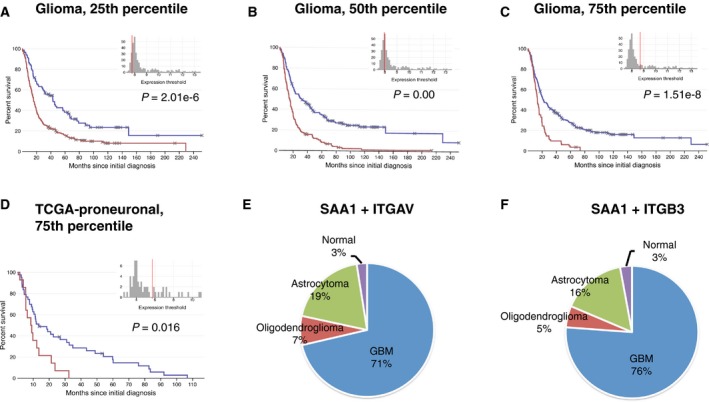
High levels of SAA1 are associated with GBM patient mortality and GBM diagnosis when integrin αVβ3 is elevated. SAA1 gene expression levels were determined to be associated with glioma patient mortality and GBM TCGA proneural subtype at different thresholds. The median level of SAA1 among patients was taken as 50th percentile, the lower quartile as 25th percentile, and the upper quartile as 75th percentile. (A–C) SAA1 gene expression levels were associated with glioma patient mortality at different thresholds (*P *<* *0.001). (D) High SAA1 level as 75th percentile was associated with mortality of patients with the TCGA proneural subtype (*P *=* *0.016). (E) Among patients exhibiting high expression levels of SAA1 and integrin αV (ITGAV), 71% had GBM; (F) among those exhibiting high expression levels of SAA1 and integrin β3 (ITGB3), 76% had GBM.

To better understand the association between SAA1 and integrin levels with GBM, we further explored the Betastasis database. The median level of SAA1 among patients was taken as 50th percentile, the lower quartile as 25th percentile, and the upper quartile as 75th percentile. SAA1 levels were associated with GBM patient mortality at 25th percentile (Fig. 5A); mortality increased with the SAA1 level (Fig. 5B,C). Patients' mortality did not differ among TCGA subtypes; however, increasing the SAA1 level sixfold (75th percentile) significantly increased the mortality of patients with the proneural subtype of GBM (Fig. 5D). Among patients with tumor integrin αV or β3 mRNA levels higher than the cutoff line (the median secretion levels in normal patients), only 45% or 39% belonged to GBM (Fig. S4A,B). Among patients with both higher levels of SAA1 and integrin αV mRNA in tumors, 71% belonged to GBM (Fig. 5E, Table S1). Among patients with higher levels of both SAA1 and integrin β3, 76% belonged to GBM (Fig. 5F, Table S2). These data indicate that increased levels of both SAA1 and integrin αVβ3 are associated with GBM.

Together, these results suggest that SAA1‐mediated glioma cell migration and invasion require cooperation with the downstream molecules integrin αV and β3. Furthermore, SAA1 together with integrin αV or β3 could be a diagnostic marker of GBM severity.

### 3.6 SAA1 enhances permeability in normal astrocytes

**Figure 6:**
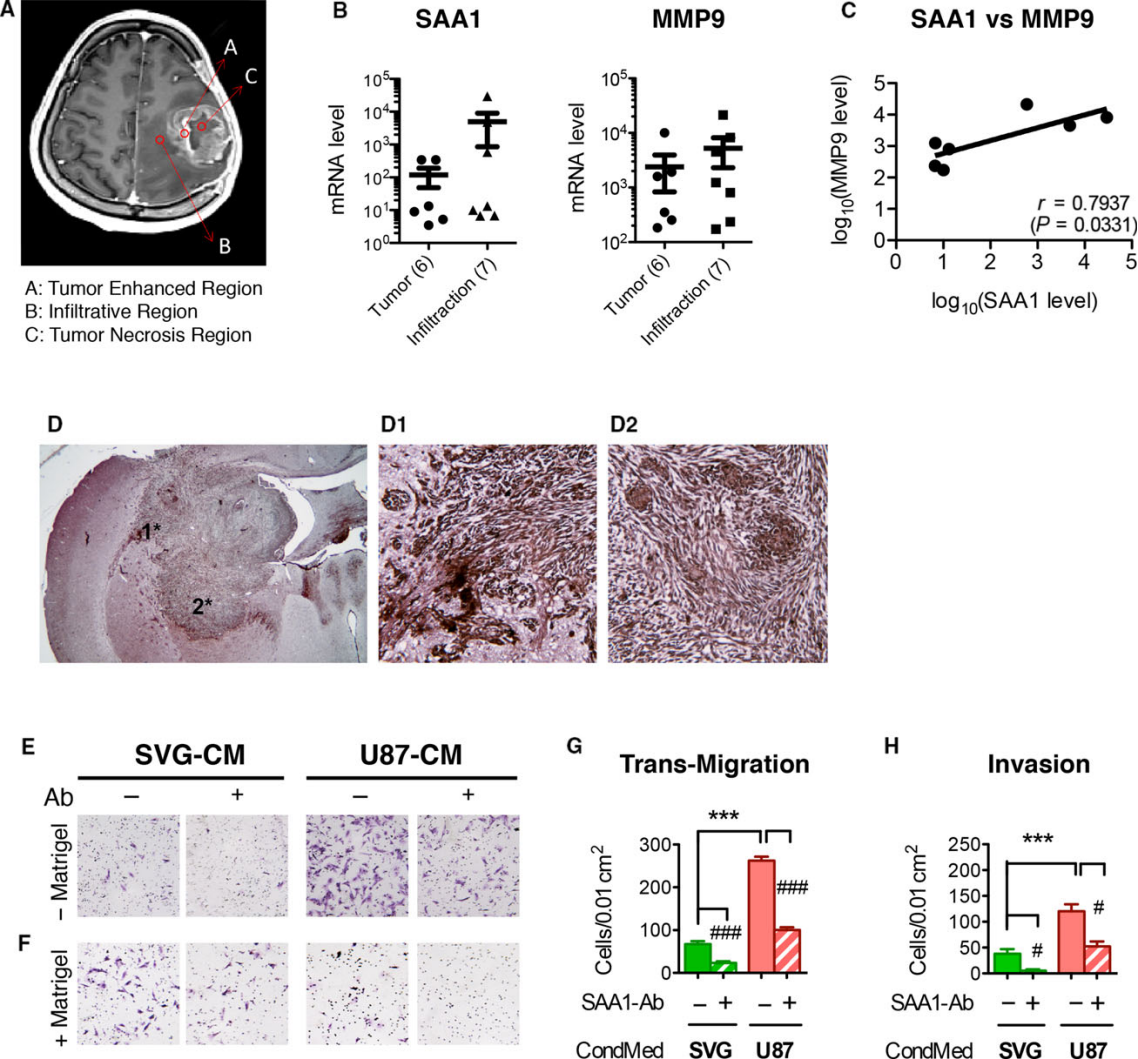
Serum amyloid A1 promotes glioma cell infiltration into normal astrocytes. (A) MRI image of a GBM tumor structure in a patient's brain (A: tumor‐enhanced region; B: infiltration region; and C: tumor necrosis region). (B) Gene expression levels of SAA1 and MMP9 in the tumor‐enhanced region and infiltration region. (C) SAA1 expression level was positively correlated with MMP9 level in the infiltration region. (D) Immunostaining of SAA1 in a GBM mouse model. Strong SAA1 immunoreactivity was discovered around the tumor; * and # mark the area enlarged in D1 and D2. (E–H) SVG cells migrated (−Matrigel) and invaded (+Matrigel) upon SVG‐CM or U87‐CM treatment in a transwell assay. Extracellular SAA1 in medium was chelated through a pretreatment with SAA1‐specific Ab (+Ab). Migration and invasion rates of SVG cells upon treatment with different media were photographed and quantified. SAA1 in CM medium enhanced the motility of normal human astrocytes, SVG cells (#*P *<* *0.05, ###*P *<* *0.001, compared with Ab‐pretreated groups. ***P <* *0.001, comparison of SVG‐CM and U87‐CM).

Glioblastoma multiforme cells infiltrating into normal brain tissue are a key indicator of glioma malignancy that is difficult to remove surgically and increases the chances of relapse after therapy. We detected the distribution of SAA1 in GBM. Different regions of GBM tissues were isolated with the help of a neurosurgeon, including a tumor‐enhanced region (region A in Fig. 6A) that contained highly proliferative cancer stem cells (CSCs), a tumor infiltration region (region B in Fig. 6A) that was suspected to have been infiltrated by tumor cells, and a tumor necrosis region in the center of the tumor (region C in Fig. 6A). A microarray was used to compare the gene profiles of different regions from brain tumor in the same patients. The SAA1 and MMP9 expression levels in region B were slightly higher than those in region A (Fig. 6B); the expression level of SAA1 was positively correlated with that of MMP9 in the infiltration region (Fig. 6C), which is consistent with our *in vitro* findings. We also detected the SAA1 distribution in a GBM mouse model; enriched SAA1 was found around the tumor infiltration region (Fig. 6D, D1, and D2). Together, these findings suggest that SAA1 is highly expressed around tumor infiltration regions rather than the CSC. In addition, we determined that extracellular SAA1 can act on normal astrocyte—SVG—migration and invasiveness. Migratory and invasive activities were low in SVG cells when they were cultured with the SVG‐CM (Fig. 6E–H). The activities increased when the cells were cultured with the U87‐CM, which had been successfully inhibited by SAA1 Ab pretreatment (U87‐CM + Ab). SVG expressed lower levels of SAA1 (Fig. 1F); blocking extracellular SAA1 in the SVG‐CM (SVG‐CG + Ab) even lowered the migratory and invasive abilities of SVG cells (Fig. 6E–H).

Together, SAA1 from GBM was determined to increase the invasion and migration of not only tumor cells but also normal astrocytes, thus promoting the infiltration of GBM cells into the normal brain region.

## 4 Discussion

This study revealed several valuable findings. First, we identified elevated SAA1 in both plasma and GBM cell secretions of patients with GBM. The level of SAA1 was associated with the glioma grade, disease severity, medication requirement, and GBM patient mortality, specially proneuronal type GBM patients. SAA1 expression was enriched in the areas surrounding tumors, which promoted GBM cell infiltration into nontumor regions. In addition, the secreted form of SAA1 was noted to increase normal astrocyte mobility. Finally, we determined integrin αVβ3 to be the key molecules responsible for SAA1‐induced GBM infiltration and the Erk signaling pathway to be the downstream mechanism. The expression levels of both SAA1 and integrin αV and β3 demonstrated a stronger association with GBM (71%–76%) compared with just SAA1 or integrin expression alone.

Our findings also reveal that plasma SAA1 can serve as a diagnostic marker of GBM severity and patient mortality. Several GBM biomarkers have been previously identified, such as PTEN, EGFRvIII, IDH1, platelet‐derived growth factor receptor A, and tumor protein 53, and they have been used to define and classify GBM subtypes by using TCGA (McNamara *et al*., 2013). Serum levels of osteopontin were reported to be correlated with a poor prognosis in patients with GBM (Sreekanthreddy *et al*., 2010), and miRNA‐326/miRNA‐130a was revealed to be highly associated with the long‐term survival of patients with GBM (Qiu *et al*., 2013). These biomarkers are genetic variants and are identified from transcriptional products that cannot be used as diagnostic markers for early detection or prognostic follow‐up. By contrast, surface markers of CSCs are favorable targets for diagnoses and may affect cancer treatment, such as CD133 for an aggressive subtype of GBM observed in younger patients with shorter survival periods (Jamal *et al*., 2012; Yan *et al*., 2011) and CD90 for high‐grade gliomas (He *et al*., 2012; Parry and Engh, 2012).

Although biomarkers of GBM stem cells have been discovered, they are still not sufficiently robust for the purpose of GBM diagnoses. Serum biomarkers are more useful for cancer risk prediction, early detection, tumor classification, and therapeutic drug monitoring such as for tumor relapse, and they can serve as therapeutic targets. Serum biomarkers, S100A8, S100A9, and CXCL4, were identified from GBM patients' serum by using surface‐enhanced laser desorption/ionization time‐of‐flight and liquid chromatography–MS/MS technologies (Popescu *et al*., 2014). Their functions are correlated with acute inflammatory reactions to cancer cells (Basso *et al*., 2014; Gebhardt *et al*., 2006) and disease‐related cystic fibrosis (van Bon *et al*., 2014; Schwartzkopff *et al*., 2009). We applied a similar proteomic methodology and found that the level of the SAA1 protein in the plasma of patients with GBM was higher than that in the plasma of healthy people; this was confirmed through protein blot analysis conducted on different cohorts. Additionally, both peripheral protein levels and genetic levels of SAA1 in tumors were significantly correlated with the malignancy of GBM and patient mortality in a dose‐dependent manner (Figs 1, 2). We also correlated this elevated plasma level of SAA1 with patients' clinical medication. We observed that patients with a higher level of SAA1 also required more aggressive medical treatment with TMZ, rather than those with a lower level of SAA1 had anti‐edema treatment. This suggests that SAA1 not only correlates with glioma grades but also highly associates with the clinical symptoms and disease severity of patients with GBM.

Our results demonstrate that SAA1 contributes to both glioma and normal astrocyte migratory and invasive abilities through the pErk signaling pathway. SAA1 knockdown was noted to reduce pErk (Fig. 3C,I). Previous studies have demonstrated that SAA1 can activate cytoskeletal rearrangement (Connolly *et al*., 2011) and focal adhesion assembly (Lung *et al*., 2015). Its receptor, FRPL1, is associated with the urokinase receptor and integrin complex (Chen *et al*., 2014; Hu *et al*., 2015), suggesting the involvement of SAA1 in cell migration signaling. In the present study, we applied two shRNA targeting SAA1 to reduce the transcription of the SAA1 protein in U87 glioma cells, which successfully reduced the motility and invasiveness of glioma cells (Fig. 3C–E). Moreover, SAA1 inhibition reduced the expression of the metastasis‐related proteins MMP9 and integrin αVβ3 in both GBM cell lines and patients' GBM cultures (Figs 3I, 4A); neutralizing the secreted form of SAA1 in medium successfully reduced glioma cell migration and invasion. Notably, we also found that extracellular SAA1 could stimulate normal astrocyte motility and invasiveness (Fig. 6E). This finding suggests that SAA1 from glioma cells serves as a paracrine to increase the mobility of normal astrocytes and facilitates cancer cell infiltration into nontumor regions, which changes the microenvironment and allows cancer cells to spread more widely.

The correlation of SAA1 with human glioblastomas was proposed in a recent study (Knebel *et al*., 2017). The study revealed that SAA1 was upregulated 10 to 60 times in patients with glioblastomas compared with patients with non‐neoplastic and other grades of astrocytoma. However, the elevation of SAA1 in serum can be directly correlated with the unfavorable outcomes for many types of cancer such as lung cancer and cervical cancer (Kim *et al*., 2015; Peng *et al*., 2015; Sung *et al*., 2011). The exact concentration of serum SAA1 that serves as the threshold for GBM diagnosis has yet to be determined. Furthermore, SAA1 can be synthesized from both the liver as an acute protein and progressive tumors; further specification of SAA1 origins (e.g., the brain) or combined detection together with other related markers could thus prove useful in GBM identification. We also identified integrin αV and β3 as the downstream signaling molecules responsible for SAA1‐induced migration of GBM cells and infiltration into normal brain tissue. Integrin αV and β3 in gliomas have been determined to be correlated with tumor grade (Gladson and Cheresh, 1991; Schnell *et al*., 2008, 2009). Integrin β3 serves as a brain‐specific organ tropism (Hoshino *et al*., 2015) that leads exosomes and vesicles targeting the brain; integrin αV together with β3 or β8 is involved in GBM disease development and progression (Gladson and Cheresh, 1991; Guerrero *et al*., 2017; Schnell *et al*., 2009). We discovered that inhibiting SAA1 expression can reduce the amount of integrin αV, integrin β3, and MMP9. In addition, we identified integrin αV and β3 as the primary molecules responsible for SAA1‐induced glia cell migration (Fig. 4E–H).

According to Knebel *et al*. (2013), the role of SAA1 in GBM migration and invasion is contradictory. They found that recombinant SAA1 increased the migratory and invasive behaviors of T98G cells but had opposite effects on A172 cells. Clarifying this contradiction, the present study provides clear evidence demonstrating that the differential requirement of SAA1 in A172 when compared with other cells is due to its low level of integrin complex (Fig. 4B–D), despite the presence of highly expressed SAA1. Reintroducing integrin αV and β3 successfully enhanced the migratory activity and response to SAA1 in A172 (Fig. 4E–H). A similar finding was noted in another patient‐derived GBM cell line, Patient 3 (Fig. 4I–M). The present study not only clarified the dual effects of SAA1 in different GBM cell lines but also identified the downstream key molecules of SAA1‐induced glioma cell migration.

Knebel *et al*. (2017) also found that serum levels of SAA1 were associated with the grades of gliomas but did not affect the clinical outcomes of patients with GBM. By contrast, we found that the gene expression levels of SAA1 in brain tumors were associated with glioma malignancy and patient mortality at threshold 25 (Fig. 5A–C), and that high levels of SAA1 were specifically associated with the mortality of patients with the proneural type (Fig. 5D). Additionally, we found a specific distribution of SAA1 in tumor microenvironments. We found enhanced SAA1 in infiltration areas surrounding tumors by performing a region‐specific microarray analysis (Fig. 6A–C) and immunostaining of SAA1 in the GBM animal tumor (Fig. 6D). Conversely, the central necrosis and CSC regions in tumors expressed lower levels of SAA1 (Fig. 6A,B,D). These findings demonstrate that the expression of SAA1 in gliomas is region dependent and could explain the inconsistent SAA1 expression in tumors versus peripheral blood and the difficulty of finding a threshold of SAA1 in GBM identification.

In addition to glioma cells, SAA1 induced the migration of normal astrocytes (Fig. 6E–H) and has been shown to promote angiogenesis in nasopharyngeal carcinoma and GBM (Knebel *et al*., 2017; Lung *et al*., 2015). Given the observation that SAA1 can be shed by tumor cells and reach the plasma, SAA1 may recruit peripheral immune cells such as macrophages and monocytes into the tumor microenvironments. Indeed, elevated SAA1 had been found to be associated with increased expression levels of the M2 macrophage marker CD163 and the monocyte marker CXCR4 in AGII‐IV/GBM patients (Knebel *et al*., 2017). Conversely, factors secreted by tumor‐associated macrophages (TAMs) and monocytes have been reported to facilitate tumor cell invasion, migration, and survival from chemo and radiotherapy (Dey *et al*., 2016; Jankowski *et al*., 2003; Yang and Zhang, 2017; Zhou *et al*., 2002). Targeting and reprogramming TAMs have thus become a novel strategy for tumor therapy (Mantovani *et al*., 2017; Noy and Pollard, 2014; Yang and Zhang, 2017; Zheng *et al*., 2017). However, research revealed the unspecific expression of SAA1 in both tumors and TAMs, which can lead to a poor prognosis in breast cancer cases (Yang *et al*., 2016).

This study demonstrated SAA1 as a molecular/metabolic signature that can help identify whether patients are at high risk of malignant GBM. Notably, we elucidated the mechanism through which SAA1 promotes astrocyte migration and invasion through integrin αVβ3. These findings serve to delineate SAA1 function as a diagnostic indicator of GBM.

## Author contributions

SYC, WCL, and TIH conducted the cellular studies, stable cell line generation, and immunostaining. YCH performed the clinical human data statistical analysis. YHC and KYC isolated and established patients GBM cell lines. FTH and CYC perform the CT scan and tissues microarray analysis from patients. JYC provided the brain slides of GBM mouse. CYL, YNP, HMS, and CCC performed the Mass Spectrometry studies and plasma analysis. STY, SCS, YKS, WLL, YSY, and YHC collected the clinical patient brain and blood samples. SYC and CCC developed the experimental design and manuscript preparation.

## Supporting information


**Fig. S1.** Representative image of serum amyloid A1 (SAA1) immunoreactivity scores.
**Fig. S2.** Cell proliferation rate and migratory activity.
**Fig. S3.** Levels of integrin αV and β3 in different GBM cells.
**Fig. S4.** Frequencies of (A) integrin αV (ITGAV) and (B) integrin β3 (ITGB3) gene expression in patients with different brain tumor grades and normal controls.
**Table S1.** Frequencies of both SAA1 and integrin αV gene expression levels among patients with brain tumors and normal controls.
**Table S2.** Frequencies of both SAA1 and integrin β3 gene expression levels among patients with brain tumors and normal controls.
