# Supplementary material for: Serum amyloid A1 in combination with integrin αVβ3 increases glioblastoma cells mobility and progression
Source: Mol Oncol. 2018 Apr 17;12(5):756–71. doi: 10.1002/1878-0261.12196 (PMC5928363; doi:10.1002/1878-0261.12196)
Supplement: Supplementary file 1 — Fig. S1. Representative image of serum amyloid A1 (SAA1) immunoreactivity scores. Fig. S2. Cell proliferation rate and migratory activity. Fig. S3. Levels of integrin αV and β3 in different GBM cells. Fig. S4. Frequencies of (A) integrin αV (ITGAV) and (B) integrin β3 (ITGB3) gene expression in patients with different brain tumor grades and normal controls. Table S1. Frequencies of both SAA1 and integrin αV gene expression levels among patients with brain tumors and normal controls. Table S2. Frequencies of both SAA1 and integrin β3 gene expression levels among patients with brain tumors and normal controls. [file MOL2-12-756-s001.docx]

**Supplementary Files**

**Serum amyloid A1 in combination with integrin αVβ3 increases glioblastoma cells mobility and progression**

Ching-Yu Lin^1#^, Shun-Tai Yang^2,3,4,5#^, Shing-Chuan Shen^6^, Yi-Chen Hsieh^7,8^, Fei-Ting Hsu^9,10,11^, Cheng-Yu Chen^9,10,11^, Yung-Hsiao Chiang^3,7,8,12^, Jian-Ying Chuang^7,8^, Kai-Yun Chen^7,8^, Tsung-I, Hsu^7,8^, Wan-Chong, Leong^7,8^, Yu-Kai Su^4^, Wei-Lun Lo^2,7,8^, Yi-Shian Yeh^4^, Yudha Nur Patria^13^, Hsiu-Ming Shih^13,14^, Che-Chang Chang^13,15^*, Szu-Yi Chou^7, 8^*

**Corresponding author:**

To who correspondence should be addressed contact should be sent to:

**Szu-Yi Chou PhD,**

The PhD Program for Neural Regenerative Medicine,

Graduate Institute of Neural Regenerative Medicine,

College of Medical Science and Technology, Taipei Medical University

E-mail: [sichou@tmu.edu.tw](mailto:sichou@tmu.edu.tw); [sichou@gmail.com](mailto:sichou@gmail.com)

Che-Chang Chang PhD,

The PhD Program for Translational Medicine,

Graduate Institute of Translational Medicine,

College of Medical Science and Technology, Taipei Medical University

E-mail: ccchang168@tmu.edu.tw

**Inventory of Supplemental Information**

1. **Supplemental Materials and Methods**
2. **Supplemental Figures and Legends**
3. **Supplemental Tables and Legends**

**1. Supplemental Materials and Methods**

***Protein concentration determination of plasma***

A Pierce® Bicinchoninic Acid (BCA) Protein Assay Kit (Thermo Scientific, Vilnius, Lithuania) was used to quantify protein concentrations. Triplicate plasma samples and bovine serum albumin (BSA) standards were added to a flat-bottomed 96-well plate which contained 100 µL of working reagent followed by incubation for 30 min at 37 °C. The absorbance was measured at 562 nm on an enzyme-linked immunosorbent assay (ELISA) reader (EZ Read 400 Microplate Reader, Biochrom, UK). A linear standard curve formula for protein quantification was made by plotting the blank-corrected BSA standard absorbance against the BSA standard concentration. Protein concentrations of samples were calculated by putting the blank-corrected sample absorbance into the linear standard curve formula.

***Immunodepletion of a highly abundant protein (albumin)***

The HSA CaptureSelect^TM^ Proteomics Depletion Product (Life Technologies, Carlsbad, CA, USA) was used to deplete albumin. Albumin immunodepletion was performed according to the manufacturer's manual. In brief, 400 µg of protein resuspended in 50 µL of phosphate-buffered saline (PBS) from each healthy subject and patient plasma protein was added to an Eppendorf tube containing 100 µL of the human serum albumin (HSA) antibody followed by incubation on a rotating shaker for 60 min at 4 °C. Afterwards, the mixture was transferred to a multi-spin separation column kit (Axygen, Carlsbad, CA, USA) and centrifuged at 12,000 rpm for 1 min at 4 °C to collect albumin-depleted plasma samples. In order to check the efficiency of albumin immunodepletion, the original samples and albumin-depleted samples were run side-by-side in sodium dodecylsulfate polyacrylamide gel electrophoresis (SDS-PAGE).

***Protein digestion***

An In-Solution Tryptic Digestion and Guanidination Kit (Thermo Scientific) was used to digest albumin-depleted samples into peptides. Protein digestion was done according to the manufacturer’s instructions. In the reduction step, 15 µL of digestion buffer, 1.5 µL of reducing buffer, and 3 µL of albumin-depleted samples were mixed. Ultrapure water was added to adjust the final volume to 27 µL followed by incubation at 95 °C for 5 min. Three microliters of alkylation buffer containing iodoacetamide was added to the mixture and incubated for 20 min in the dark at room temperature. In the digestion step, 1 µL of activated trypsin was added to the mixture, followed by incubation for 3 h at 37 °C. We added an additional 1 µL of activated trypsin to the mixture (with a final volume of 32 µL) and re-incubated the reaction overnight at 30 °C.

***ZipTip C18 procedures***

The purpose of the ZipTip procedure was to remove salts which might interfere with the mass spectrometric (MS) analysis. The pH of tryptic-digested samples was adjusted to <4 by titrating digested samples with 1% or 10% trifluoroacetic acid (TFA) in order to stop the trypsin digestion reaction. For each sample, six Eppendorf tubes were prepared which contained the following solutions: (1) tube 1 had 1 mL of acetonitrile (ACN)/0.1% TFA; (2) tube 2 had 0.5 mL ACN/0.1% TFA plus 0.5 mL H_2_O/0.1% TFA; (3) tube 3 had 1 mL H_2_O/0.1% TFA; (4) tube 4 was empty (used for waste after washing); and (5) tubes 5 and 6 each contained 30 µL of tube 2 solution. The ZipTip C18 tip (ZTC18S960, Merck Millipore, Billerica, CA, USA) was equilibrated by pipetting 20 µL of solution into tubes 1 to 3. Once it was equilibrated, the tip was used to capture the peptides contained in the tryptic-digested sample solution by pipetting the solution up and down 15 times. Repeated aspiration-dispensing (5~15 times) of the tube 3 solution was done to wash the captured peptides. The aspirated solution was then dispensed into tube 4. The captured peptides were eluted by pipetting the solution up and down in tubes 5 and 6 times. The eluted peptides were dried in a centrifugal vacuum concentrator (SpeedVac SC110 Concentrators, Savant Instrument, Holbrook, NY, USA) for 60 min.

***Liquid chromatography tandem MS (LC-MS/MS) conditions***

LC was performed on a NanoAquity UPLC system (Waters, Milford, MA, USA) coupled to an Orbitrap Elite mass spectrometer (Thermo Electron, Waltham, MA, USA). Peptide samples were injected into a trap column (2 cm × 180 μm i.d., Symmetry C18, Waters), and then were separated by a 25-cm × 75-μm-i.d. BEH130 C18 column (Waters) with a segmented gradient in 120 min from 0% to 85% solvent B at 300 nL/min (buffer A, 0.1% formic acid in H_2_O; buffer B, 0.1% formic acid in ACN). The Orbitrap was operated in the positive ion mode, with the following acquisition cycle: a full scan (m/z 350~1600) recorded in the Orbitrap analyzer at resolution R = 240,000 was followed by MS/MS of the ten most intense peptide ions in the ion trap analyzer. Peptide fragmentation by collision-induced dissociation was automatically performed in a dynamic data-dependent mode. All measurements in the Orbitrap were performed with the lock mass option to improve the mass accuracy of precursor ions.

***Protein database search and protein identification***

All Orbitrap Elite MS raw data files were qualitatively and quantitatively processed using PEAKS Studio (PEAKS 7, Bioinformatic Solution, Ontario, Canada). Settings for the protein database search were as follows: the protein database was the Uniprot-Human with a decoy database; the enzyme was trypsin with a maximum of two missed cleavage sites; the precursor mass tolerance was 20 ppm; the fragment mass tolerance was 0.8 Da; and the false discovery rate (FDR) value was <1%. Post-translational modifications (PTMs) were matched with 485 types of PTMs available in the database. Detected peptide-spectral matches (PSMs) were additionally filtered with the following criteria: a peptide score of ≥20 (in the form of -10logP), a multiple of change of ≥1, and unique peptides of ≥1.

***Label-free quantification (LFQ) of peptide spectra***

Protein intensity quantification was done using an LFQ method. PEAKS 7 uses an expectation-maximization (EM)-based algorithm for feature detection, deconvolution, and refinement. An optimization model for simultaneous feature matching and retention time alignment was used in the analysis. The LFQ parameters used were: a mass error tolerance of 20 ppm; a retention time shift tolerance of 6 min; and an FDR value of <1%. Peptide features and proteins with multiples of change of ≥2.0 and a statistical *p* value of <0.05 were considered significant between patients with [GC/GBM] and healthy controls (HCs). Experimental bias was taken into account by automatic normalization of protein ratios based on the total ion chromatogram (TIC).

**2. Supplementary figures and legends**

**
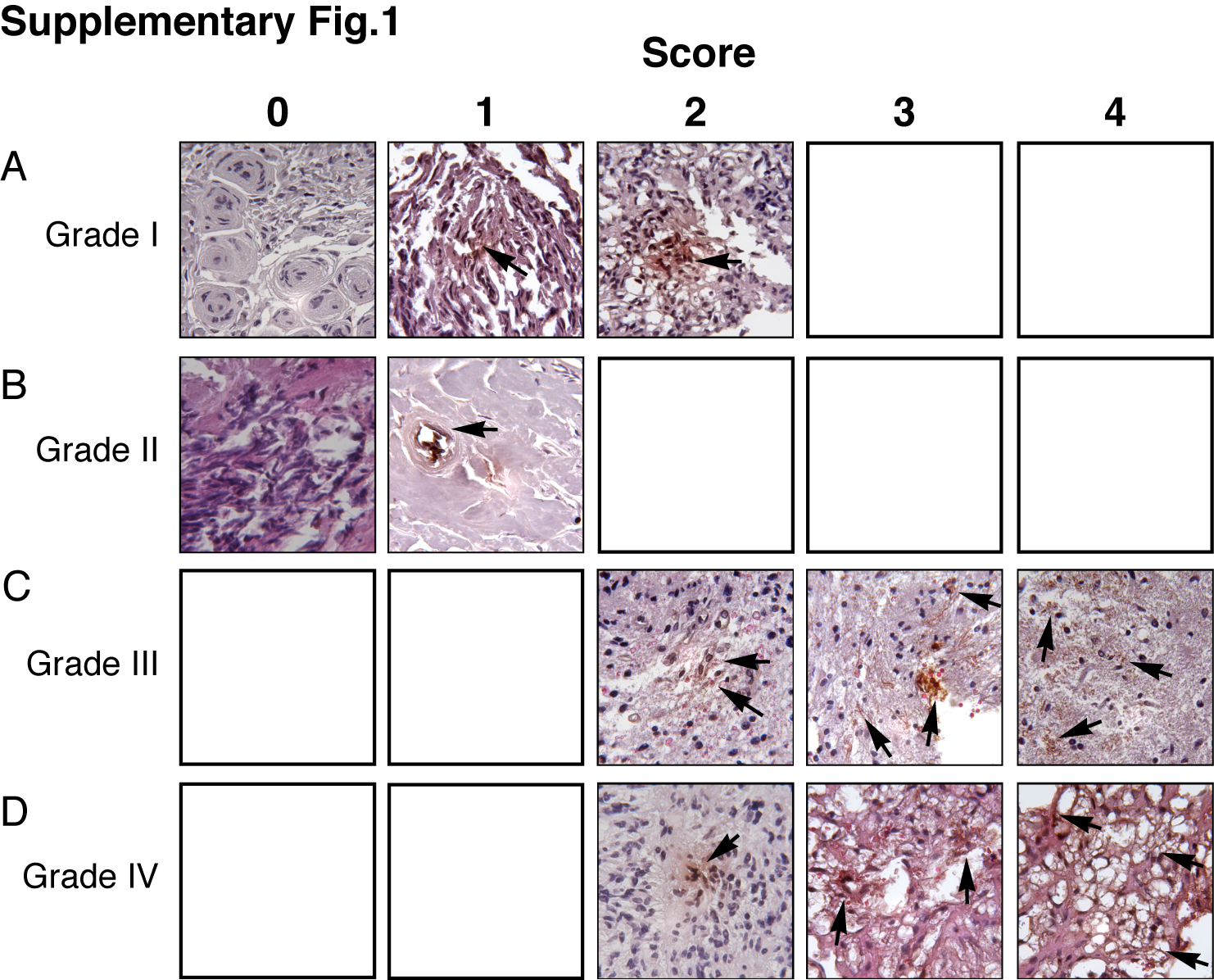
**

**Fig. S1. Representative image of serum amyloid A1 (SAA1) immunoreactivity scores.** Representative images of SAA1 immunoreactivity scores of 0–2 in glioma grades (A) I and (B) II and scores of 2–4 in glioma grades (C) III and (D) IV.


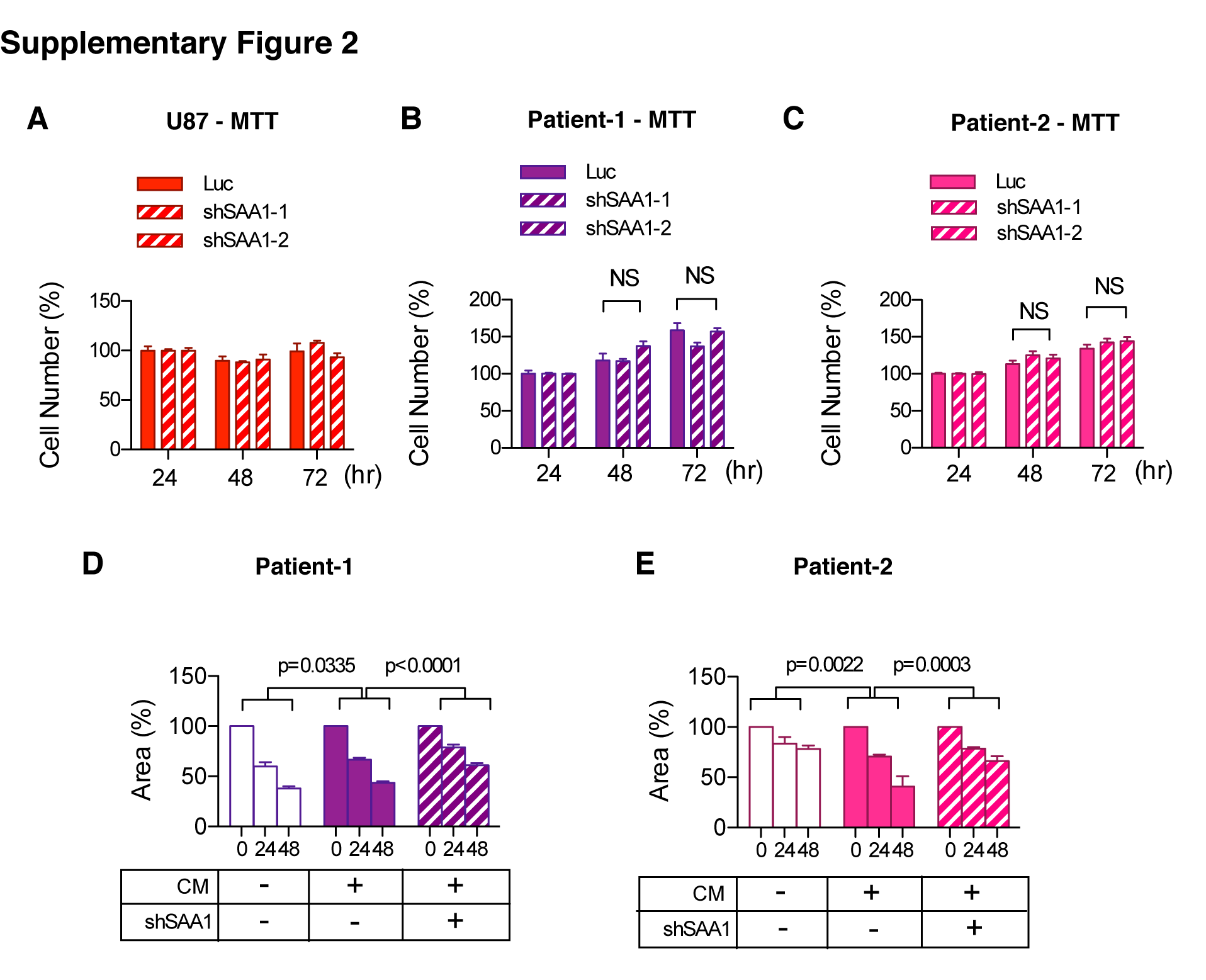


**Fig. S2. Cell proliferation rate and migratory activity.** The proliferation of U87MG (U87) and patient-derived glioblastoma multiforme (GBM) cells—Patients 1 and 2—was analyzed through the MTT assay. The proliferation rate did not change after the silencing of SAA1 in (A) U87, (B) Patient 1, and (C) Patient 2 cells. The migratory ability of Patient 1 and 2 cells treated with serum-free medium, conditioned medium (CM), or SAA1-Ab pretreatment (+Ab) was analyzed through a wound-healing assay. Migration areas between cells were measured and quantified after 0, 24, and 48 h. (D, E) Migration rate was increased when cells were treated with CM, which was reduced in SAA1-pretreated cells (* *p* < 0.05; ** *p* < 0.01; *** *p* < 0.001, compared with short-hairpin RNA scrambled nucleotides through two-way ANOVA).


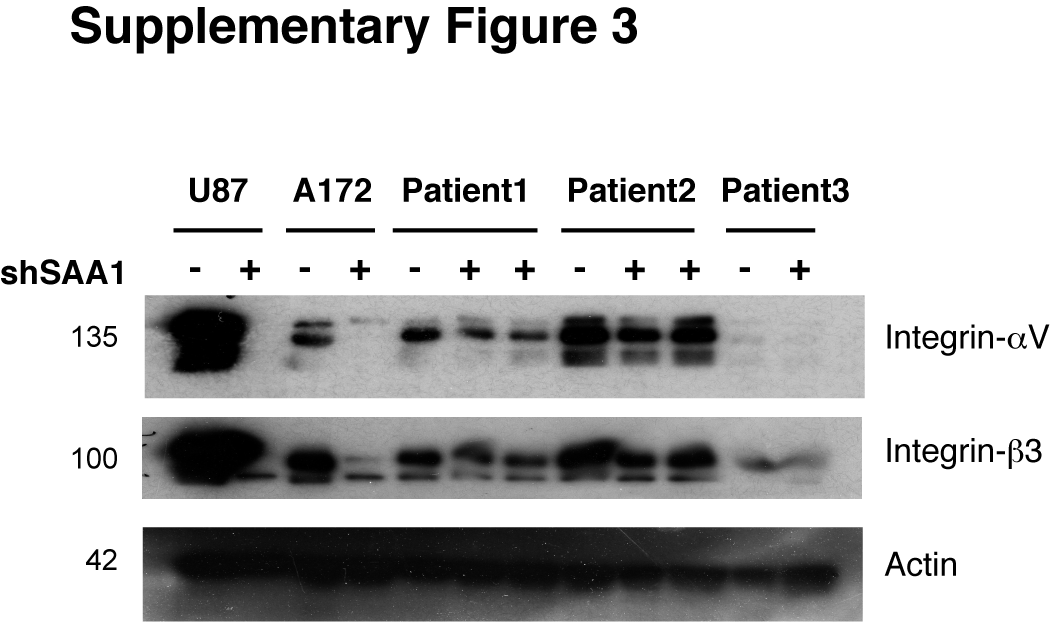


**Fig. S3. Levels of integrin αV and β3 in different GBM cells.** The protein levels of integrin αV and β3 in U87, A172, and three different patients’ GBM cell lines were analyzed by Western blotting. A172 and Patient 3 GBM cells contained lower amounts of integrin αV and β3 than U87 and the other two patient-derived GBM cells. Silencing SAA1 through short-hairpin RNA also reduced the cell integrin αV and β3 levels. Actin was used as an internal control.

**
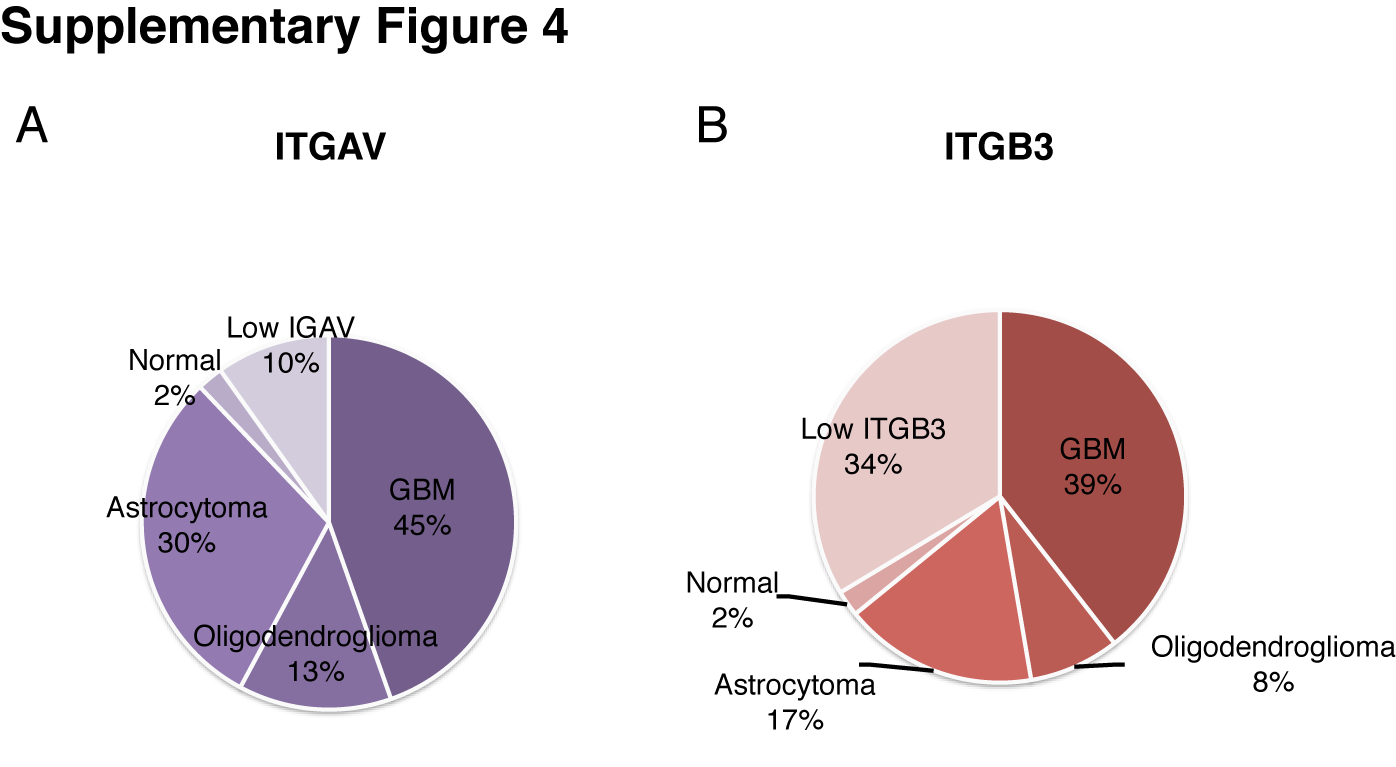
**

**Fig. S4. Frequencies of (A) integrin αV (ITGAV) and (B) integrin β3 (ITGB3) gene expression in patients with different brain tumor grades and normal controls.** Approximately 10% of patients expressed low levels of integrin αV; 2% of normal controls, 30% of patients with astrocytomas, 13% of patients with oligodendrogliomas, and 45% of patients with GBM expressed high levels of integrin αV. Low and high gene expression levels were defined according to the mean of each gene in normal controls. For integrin -β3, 34% of patients expressed low levels; 2% of normal, 17% of patients with astrocytomas, 8% of patients with oligodendrogliomas, and 39% of patients with GBM expressed high levels of integrin β3.

**3. Supplementary tables and legends**

**Table S1. Frequencies of both SAA1 and integrin αV gene expression levels among patients with brain tumors and normal controls**

| **SAA1/ITGAV** | **GBM** | **Oligodendroglioma** | **Astrocytoma** | **Normal** | **Sum** | **p-value** |
| --- | --- | --- | --- | --- | --- | --- |
| **Low/Low** | 5 | 5 | 9 | 7 | 26 | <0.0001 |
| **Low/High** | 57 | 45 | 96 | 5 | 203 |  |
| **High/Low** | 10 | 2 | 2 | 4 | 18 |  |
| **High/High** | 142 | 14 | 38 | 5 | 199 |  |

**Low and high gene expression levels were defined according to the mean of each gene in normal controls.**

**Table S2. Frequencies of both SAA1 and integrin β3 gene expression levels among patients with brain tumors and normal controls**

| **SAA1/ITGB3** | **GBM** | **Oligodendroglioma** | **Astrocytoma** | **Normal** | **Sum** | **p-value** |
| --- | --- | --- | --- | --- | --- | --- |
| **Low/Low** | 17 | 24 | 57 | 7 | 105 | <0.0001 |
| **Low/High** | 45 | 26 | 48 | 5 | 124 |  |
| **High/Low** | 21 | 7 | 13 | 4 | 45 |  |
| **High/High** | 131 | 9 | 27 | 5 | 172 |  |

**Low and high gene expression levels were defined according to the mean of each gene in normal controls.**
